# Insights into substrate binding of ferulic acid esterases by arabinose and methyl hydroxycinnamate esters and molecular docking

**DOI:** 10.1038/s41598-017-17260-x

**Published:** 2017-12-11

**Authors:** Cameron J. Hunt, Io Antonopoulou, Akshat Tanksale, Ulrika Rova, Paul Christakopoulos, Victoria S. Haritos

**Affiliations:** 1 Department of Chemical Engineering, Monash University, Clayton, 3800 Victoria Australia; 2 Department of Civil, Environmental and Natural Resources Engineering, Luleå University of Technology, Luleå, Sweden

## Abstract

Ferulic acid esterases (FAE, EC 3.1.1.73) cleave the arabinose hydroxycinnamate ester in plant hemicellulose and other related substrates. FAE are commonly categorised as type A-D based on catalytic activities towards model, short alkyl chain esters of hydroxycinnamates. However, this system correlates poorly with sequence and structural features of the enzymes. In this study, we investigated the basis of the type A categorisation of an FAE from *Aspergillus niger*, AnFaeA, by comparing its activity toward methyl and arabinose hydroxycinnamate esters. k_cat_/K_m_ ratios revealed that AnFaeA hydrolysed arabinose ferulate 1600-fold, and arabinose caffeate 6.5 times more efficiently than their methyl ester counterparts. Furthermore, small docking studies showed that while all substrates adopted a catalytic orientation with requisite proximity to the catalytic serine, methyl caffeate and methyl *p-*coumarate preferentially formed alternative non-catalytic conformations that were energetically favoured. Arabinose ferulate was unable to adopt the alternative conformation while arabinose caffeate preferred the catalytic orientation. This study demonstrates that use of short alkyl chain hydroxycinnnamate esters can result in activity misclassification. The findings of this study provide a basis for developing a robust classification system for FAE and form the basis of sequence-function relationships for this class.

## Introduction

Ferulic acid esterases (FAE, EC 3.1.1.73) cleave the ester linkage between hydroxycinnamate and the C(O)5 position of an arabinose residue in hemicellulose, a plant-derived sugar polymer. This linkage is referred to as FAX (2-*O*-[5-*O-trans*-feruloyl)-*β*-L-arabinofuranosyl]-D-xylopyranose) and it is the primary covalent interaction between lignin and hemicellulose^1^ which, of themselves, are two of the three main polymers made by plants. FAE have received much recent attention for their potential use in industrial transformations; the substrate promiscuity of some FAE has been employed for the deconstruction of plant biomass for biofuels and animal feed applications^2,3^ and for transesterification reactions utilising hydroxycinnamic esters^4^.

**Figure 1:**
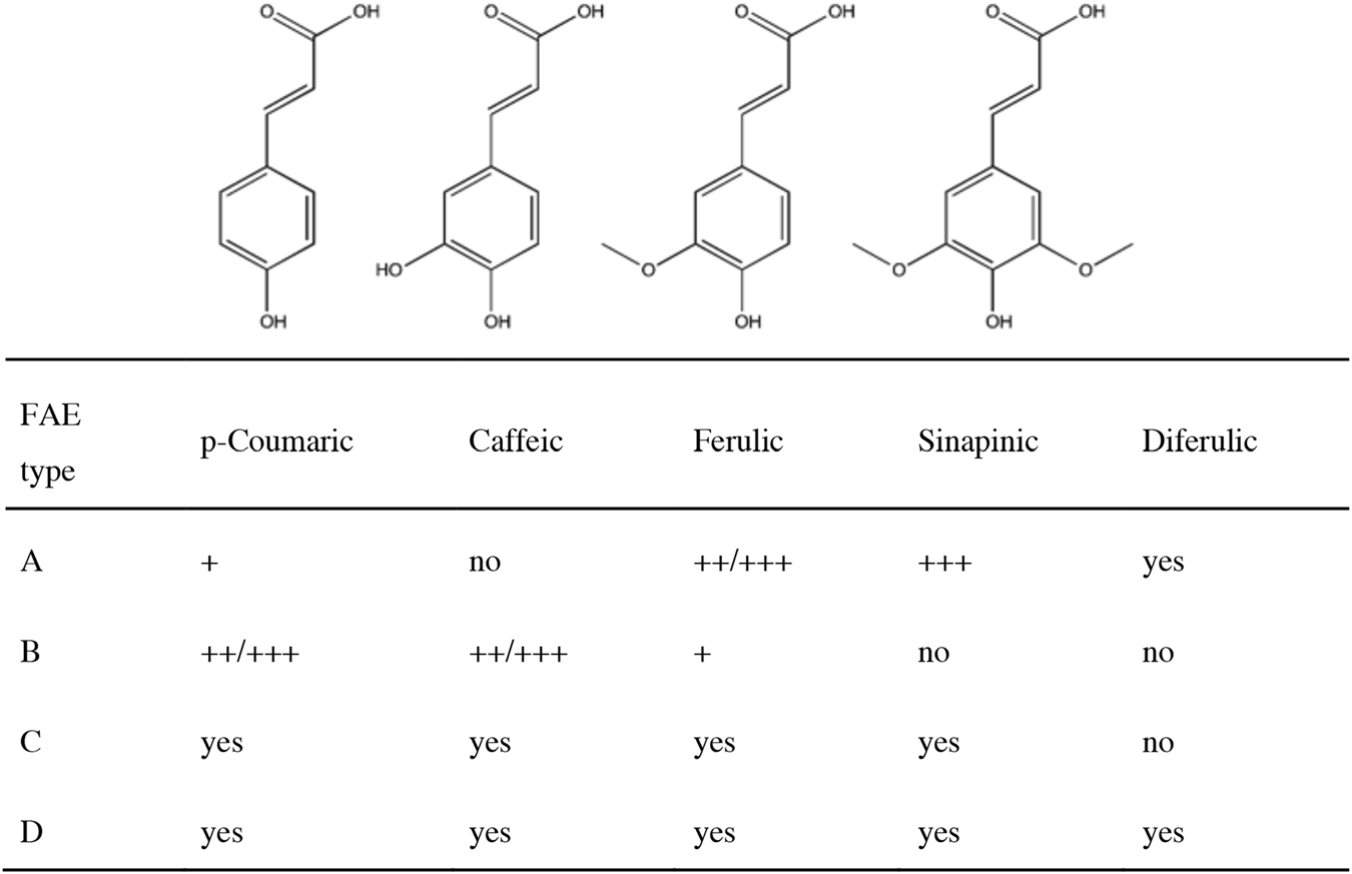
Classification of FAE according to the system developed by Crepin *et al*.^7^. Reproduced in part from Kuhnel *et al*.^33^.

As the degree of reactivity of different FAE towards hydroxycinnamic ester linkages in biomass has become important in itself and valuable for their commercial applications, classification systems based on substrate specificity have been developed for the enzyme family. The most common hydroxycinnamic acids found in plant biomass are ferulic, *p-*coumaric, caffeic and sinapinic^5^ which vary in the number of hydroxy and/or methoxy substituents in the meta-positions of the aromatic ring (Fig. 1). One of the leading classification systems for FAE is based on the ability of a candidate to hydrolyse model substrates, *viz* the methyl- or ethyl esters of hydroxycinnamic acids such as ferulate (MFA/EFA), methyl *p-*coumarate (MpCA), methyl caffeate (MCA) and methyl sinapate (MSA). Initially, FAE candidates were divided into either type A or B depending on their activity towards MSA or MCA respectively^6^, and this was based on the activities of two classical FAE, AnFaeA and AnFaeB, derived from *Aspergillus niger*. The type A/B classification was later extended to include type C and D FAEs^7^. In general, type A FAEs show increased activity against model phenolic substrates containing a methoxy substitution at carbon-3 or -5, such as MSA and MFA. Type B FAE prefer small polar or no substitutions at carbon-3 or -5 and thus have strong activity towards MCA and MpCA. Type C and D are active against all of these model short chain hydroxycinnamic esters but differ in their ability to hydrolyse esters of diferulic acid. The FAE type A-D classification system is summarised in Fig. 1.

Genome sequencing programs have generated a large diversity of putative FAE sequences from bacteria and fungi and it would be very valuable to be able to build amino acid sequence-activity relationships for this expanding class of enzymes. However, attempts to link the type A-D activity classification system, which is based on hydrolysis of short chain esters of hydroxycinnamic acids, and the amino acid sequences of biochemically characterised FAE have hitherto been unsuccessful, resulting in groupings that contain mixed FAE activity types^7–9^. For example, Benoit *et al*. categorised fungal FAE into 7 sub families which were later extended to 13 groupings based on phylogenetic analysis^10,11^. Subfamily 1 contained both B and C FAE, including the well characterised FAE, AnFaeB, as well as FAE from *Aspergillus oryzae*, AoFaeB and AoFaeC. The difficulty in aligning activity and sequence may be due, in part, to the diversity of the FAE enzyme class, with some putative members having close sequence similarity to lipase, acetyl xylan esterase, xylanase and chlorogenate esterase enzymes. Regardless, our ability to predict the activity of a novel FAE based on sequence similarity to a characterised FAE is limited.

**Figure 2:**
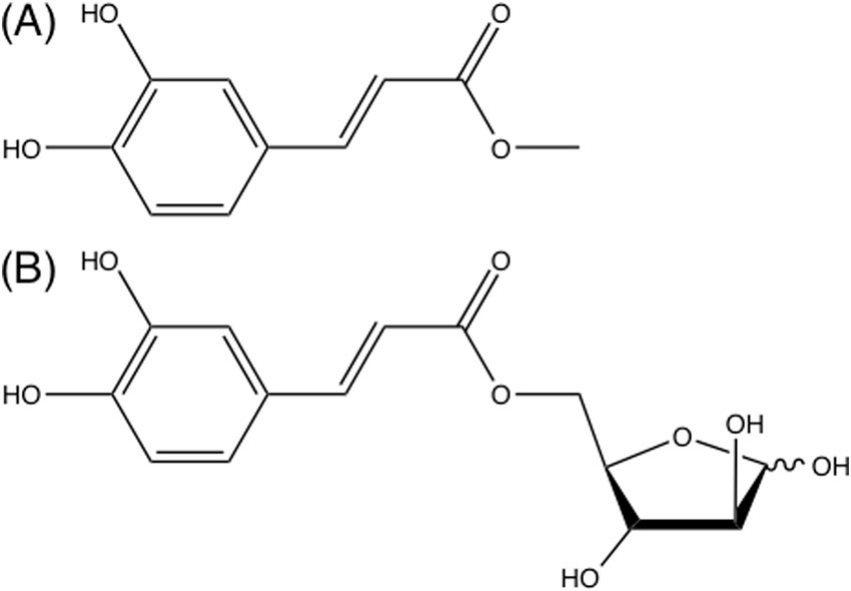
(**A**) Structure of methyl caffeate (MCA) and (**B**) structure of arabinose caffeate ester (ACA).

Enzyme classification is a useful tool in reducing the complexity of whole enzyme families and their applications, but may also be a basis for sequence, structure and function relationships for that enzyme group. Classification systems can be a valuable basis for rational design of enzymes as catalysts in biotechnological applications and extensive databases have been created to support these purposes including BRENDA^12^ and CAZy^13^. While classification can take many forms including structural or sequence similarity, substrate specificity and activity are important factors and thus the selection of substrates as a basis for classification should be sound. As described above, the most commonly used FAE classification system is based on enzyme activity toward model substrates, which, while similar to the enzyme’s natural substrates of sugar-hydroxycinnamate linkages present in plant biomass, differ mostly in the alcohol component of the ester. In the natural substrate, the alcohol is a pentose monosaccharide such as arabinose, while the model esters used for activity-based classification have short chain alkyl groups, especially methyl. Structures of two caffeate ester substrates are compared in Fig. 2.

Alternatives to the FAE type A-D classification system may be more revealing in terms of understanding substrate binding and more robust in terms of classification based on enzyme activity using more diverse substrates. Molecular modelling tools such as small molecule docking (SMD) have been used to investigate the activities of FAE toward short chain ester substrates. In particular, research by Suzuki *et al*.^14^ determined the crystal structure of one of the most studied FAE obtained from *Aspergillus oryzae*, AoFaeB, and used AutoDockTools (ADT) to simulate docking of the 4 short chain ester model substrates into the active site. The researchers were able to compare the active centre and docking to that of the related *A. niger* enzyme, AnFaeA, concluding that the residue TYR356 was most likely responsible for the severely reduced activity of AoFaeB toward MFA and MSA through steric hindrance to the larger methoxy group^14^. Furthermore, Goldstone *et al*. used GOLD to predict the binding mode of ferulic acid and a feruloyl arabinose ester to a previously unknown FAE, Est1E, identified from a ruminant bacterium^15^. Correct binding orientations for hydrolytic activity were modelled for feruloyl arabinose ester and extrapolated for short chain model esters; enzyme assays verified the enzyme’s activity toward ethyl ferulate and ferulic-containing hemicellulose fractions.

In terms of the synthesis of more diverse substrates to assist in the classification of FAE by activity, FAE themselves have been used to synthesise a variety of sugar esters via transesterification, or reverse hydrolysis, in non-conventional media such as organic solvents. Some of the synthesised esters are closer in structure to the linkages that FAE hydrolyse in plant biomass such as phenolic acid sugar esters prepared by Vafiadi *et al*. 2007 and Kelle *et al*.^16,17^ compared with model substrates. Thus with both molecular modelling tools and more authentic enzyme substrates available, alternative approaches to FAE classification can be explored.

Here we compare the catalytic activities of the type A FAE from *A. niger*, AnFaeA, toward the model hydroxycinnamic acid substrates, methyl ferulate and -caffeate, and their arabinose ester counterparts. The arabinose ester substrates were synthesised by transesterification and were fully characterised. AnFaeA was specifically chosen as this was the main enzyme for which the A-D classification was based on. Furthermore, the type A category is the most robustly defined. The second most relevant enzyme to the A-D classification, AnFaeB from *A. niger*, which was initially categorized as type B has since been re-categorised as belonging to in a family of type C FAE due to its sequence similarity^11^. This goes to further highlight the complexity of the current classification systems based on enzymatic activity. In addition to its prominence in the classification of FAE, AnFaeA is a suitable candidate for the focus of this manuscript as it has a large body of literature defining its substrate specificity, has a detailed solved crystal structure and is highly similar to many of the other type A FAE. A major contributing factor to the selection of AnFaeA is the small number of significantly distinct FAE with solved crystal structures. The Protein Database (PDB) contains only 10 records under EC of 3.1.1.73 (feruloyl esterase) with only approximately three other listed outside this set. Of these AnFaeA has the most significant volume of literature relating to activity with a clear distinction of no activity on methyl caffeate. As such, we propose that the primary assessment of enzymatic characterization should be done on AnFaeA.

While an enzyme from type A such as AnFaeA would be expected to demonstrate good activity toward ferulate but negligible activity toward caffeate esters, we found this was only true for methyl esters; both arabinose hydroxycinnamic acid esters were good substrates for the enzyme. The basis for the stark contrast in enzyme activities measured for the methyl and arabinose-based esters was explored further by SMD and this provided new insights into substrate binding to FAE. The findings of this study will aid in developing a robust classification system for FAE and form the basis of a structure-function relationship for this enzyme class, whereby features of FAE sequence, structure and activity are aligned.

## Results and Discussion

### AnFaeA shows higher apparent affinity for arabinose than methyl esters of ferulic and caffeic acid

AnFaeA exhibited activity toward all methyl and arabinose esters of ferulic and caffeic acid as shown in Table 1, however, there was a 63-fold increase in the specific activity of AnFaeA towards AFA compared to MFA, and a 12-fold improvement towards ACA compared to MCA. Thus, there is a clear preference shown by AnFaeA towards arabinose- compared with methyl esters. This preference has been shown in earlier work, where the FAE from *Clostridium thermocellum* and *Neocallimastix sp*. had increased activity toward FAXX and PAXX (2-*O*-[5-*O-trans*-feruloyl)-*β*-L-arabinofuranosyl]-(1,3)-*O*-*β*-D-xylopyranosyl-(1,4)-D-xylopyranose and 2-*O*-[5-*O-trans-p*-coumaroyl)-*β*-L-arabinofuranosyl]-(1,3)-*O*-*β*-D-xylopyranosyl-(1,4)-D-xylopyranose respectively) over MFA^18,19^.

**Table 1:** Specific activity of AnFaeA towards the methyl and arabinose esters of ferulic and caffeic acid.

Ester substrates	Specific activity (*μ* mol free acid/(mg FAE. min))
Ferulate	
MFA	17.3 ± 1.7
AFA	1084.0 ± 151.3
Caffeate	
MCA	0.24 ± 0.06
ACA	2.83 ± 0.42

In terms of hydroxycinnamate linkage, ferulate esters were better substrates than caffeate for AnFaeA which is in agreement with the current classification system^7^, that is, if restricting the discussion to methyl esters, the activity toward caffeate was almost undetectable (72-fold lower) which is the main characteristic of type A FAE specificity. By contrast, the activity of AnFaeA against ACA was only 6-fold lower than MFA activity showing the enzyme can also hydrolyse caffeate esters at reasonable rates, which is usually characteristic of type B, C and D FAEs.

**Figure 3:**
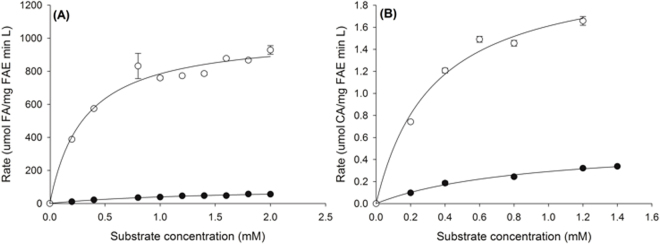
Velocity versus substrate concentration curves comparing the hydrolysis rates of arabinose and methyl esters of hydroxycinnamic acids (**A**) AFA (open circles) and MFA (solid circles) (**B**) ACA (open circles) and MCA (solid circles). Values are shown as the mean and s.d. for duplicate experiments.

The enzyme velocity versus substrate concentration curves for AnFaeA towards the methyl and arabinose esters of hydroxycinnamic acids are shown in Fig. 3 and demonstrate the stark differences in velocity of the enzyme reaction towards esters with a short alcohol or sugar substituent attached to the same acid group. The kinetic parameters (kcat and K_m_) were determined from the fit of the Michalis-Menten reaction to the data (Table 2); AnFaeA shows an apparent higher affinity for arabinose esters than methyl esters, as the estimated K_m_ is 5-fold lower for AFA than MFA, and 3 times lower for ACA than MCA. In general, AnFaeA shows higher affinity towards MFA than MCA, as expected under the current classification system. However, the affinity towards the equivalent sugar-linked pair, AFA and ACA, is almost the same (0.31 and 0.33 mM, respectively) revealing an important difference in AnFaeA affinity towards sugar-linked hydroxycinnamate esters to that of model substrates. The k_cat_/K_m_ ratio revealed that AnFaeA hydrolyses AFA 1600 times more efficiently than MFA, and ACA 6.5 times more than MCA. The extremely high k_cat_/K_m_ ratio for AFA is attributed to the very fast reaction (high k_cat_) and the high substrate affinity (low K_m_) for this substrate.

**Table 2:** Kinetic parameters of AnFaeA determined against the methyl and arabinose esters of ferulic and caffeic acid.

Ester	V_max_ (*μ*moles/(mg FAE. min. L))	K_m_ (mM)	k_cat_ (1/*m* *g FAE*. *sec*)	k_cat_/K_m_ (1/*m* *g FAE*. *sec*. *mM*)
Ferulate				
MFA	96.4 ± 9.0	1.42 ± 0.26	132.5	93.2
AFA	1027.9 ± 46.6	0.31 ± 0.06	47,089.6	151,657.3
Caffeate				
MCA	0.55 ± 0.05	0.87 ± 0.17	0.05	0.067
ACA	2.14 ± 0.16	0.33 ± 0.07	0.14	0.439

**Figure 4:**
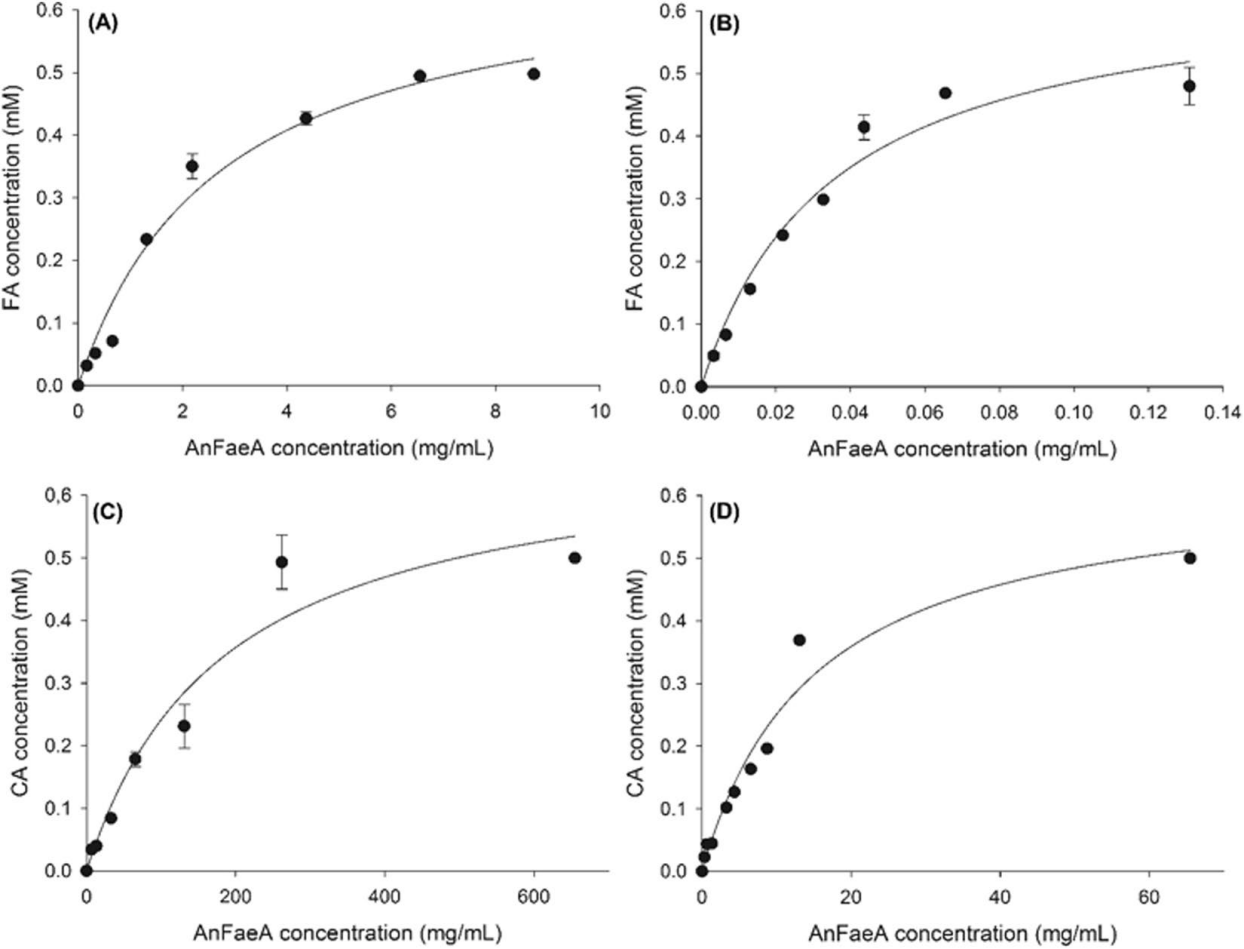
Effect of AnFaeA protein concentration on hydrolysis of arabinose and methyl esters of hydroxycinnamates (**A**) MFA (**B**) AFA (**C**) MCA (**D**) ACA.

The characterisation of AnFaeA shows that the enzyme has greater activity on substrates containing a more bulky sugar linkage, such as AFA and ACA, than the respective short alkyl chain ester. Generally, type A FAEs show highest activity against phenolic substrates containing a methoxy substitution at C-3 and C-5 such as MFA and MSA while no activity has been detected for MCA according to previous reports^9^. Nevertheless, in the present study a low level of activity was detected with MCA which is attributed to the high enzyme load used for the MCA assays, approximately 100 times higher than the one used for MFA. The comparative protein concentrations for ester hydrolysis are shown in Fig. 4 for the 4 substrates. In studies with type A enzymes where MFA or MSA activity is being assessed, activity on MCA would not normally be detected. Accordingly, at enzyme loads suitable for MFA, satisfactory activity could be also measured with ACA whereas no activity was detected with MCA (Table 2).

From the characterisation of AnFaeA on both MFA and AFA it is clear that the moiety attached via the ester linkage can have a significant effect on the catalytic activity of the FAE. This observation is supported by studies conducted by Topakas *et al*. and Vafiadi *et al*.^20,21^ who used various alkyl ferulates to characterise FAE. In those reports, FAE activity was detected against ferulic acid esters ranging from methyl to butyl. Interestingly, the k_cat_/K_m_ of MtFae1a towards ethyl ferulate was less than half of the same measure for either methyl ferulate or n-propyl ferulate. Other studies have shown FAE with activity towards esters of *p-*nitrophenyl or alpha-naphthyl of up to C8 in length^22,23^. However, while some attention has been drawn towards the effect of the alcohol-side constituent of the ester group on FAE activity, its influence when combined with a range of hydroxycinnamic acids has been overlooked. Thus an explanation for the large difference in activity demonstrated by AnFaeA towards MCA and ACA is not currently forthcoming.

### Docking of small hydroxycinnamic esters to AnFaeA reveals an alternative energetically favourable binding orientation

**Figure 5:**
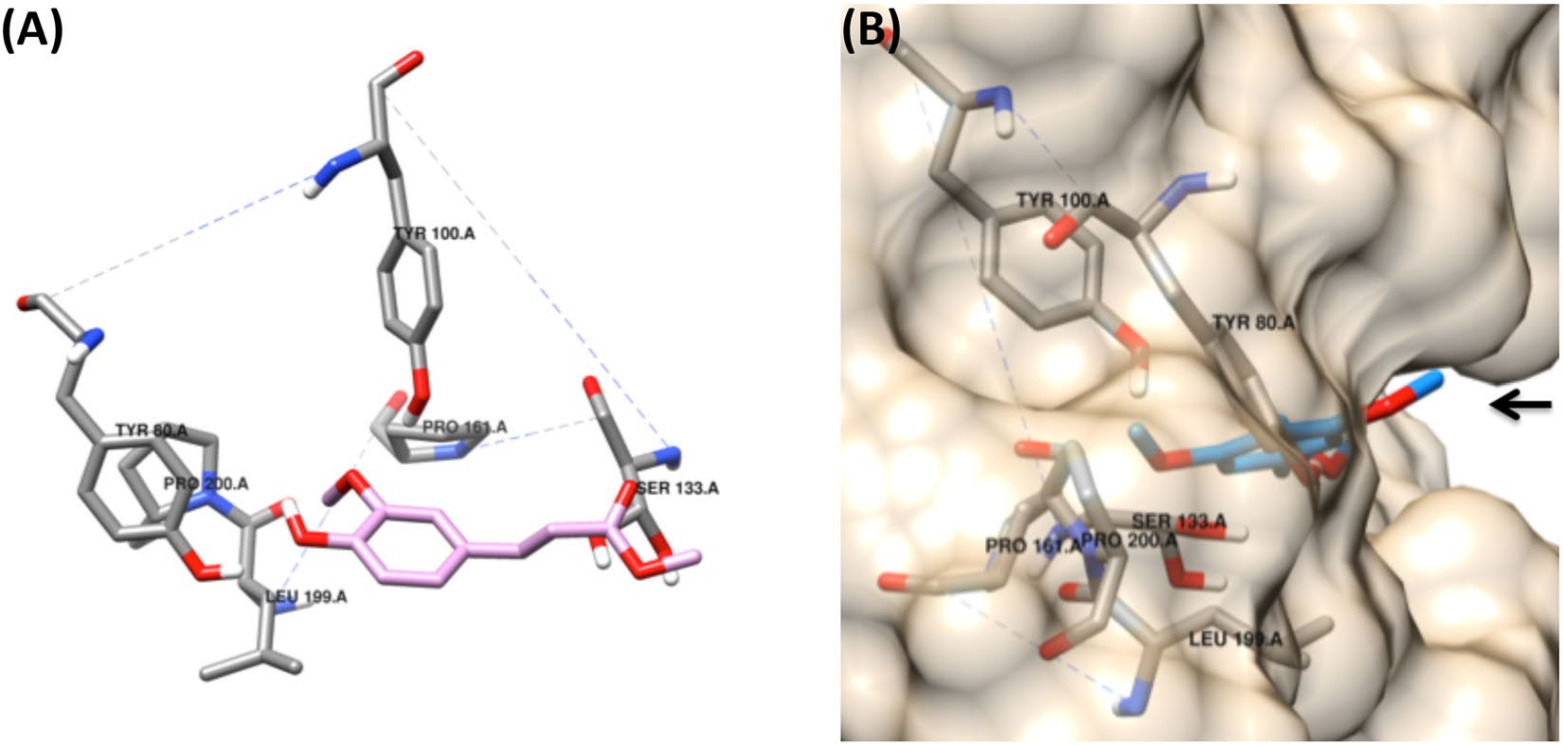
(**A**) Binding of MFA into the active site of AnFaeA. The residues responsible for maintaining the hydrophobic hole are indicated as is the catalytic serine based on Faulds *et al*.^9^ and simulated using AutoDock tools. (**B**) Binding of MSA into the active site of AnFaeA in the only conformation that is catalytically active via docking simulations. The second methoxy group (indicated by the arrow) is pointed away from the binding site and into the solvent space and having less interaction with AnFaeA.

Investigation of the AnFaeA protein structure by Faulds *et al*.^9^ revealed that the methoxy residue of ferulic acid occupies a hydrophobic hole in the binding cavity of the enzyme (PDB: 1UZA) which is made from the residues PRO161, PRO200, ILE199 and TYR80 as shown in Fig. 5A. For sinapinic acid which has two opposing methoxy residues in positions 3- and 5- of the phenolic ring one methoxy residue occupies the hydrophobic hole in the binding cavity while the second extends outwards into the solvent with less interaction with AnFaeA (Fig. 5B). These orientations of ferulic and sinapinic acids within the active site of AnFaeA were used in the current work to extrapolate the conformations of the other hydroxycinnamic acids in consideration such as *p-*coumarate and caffeate.

While AnFaeA is a type A FAE with strong activity towards MFA and MSA, there is also detectable activity toward MpCA but no MCA activity recorded^9^. Simulated docking of hydroxycinnamic esters was undertaken with the enzyme to investigate the basis for lack of activity of the closely related structures. The conventional reasoning has been that a polar hydroxyl side group of the phenolic ring of the hydroxycinnamic acid cannot occupy the hydrophobic hole^9^. However, with the knowledge that one methoxy group of sinapinate points outwards from the enzyme (Fig. 5B), it is also possible for the equivalent group in caffeate, a hydroxyl, to adopt this same orientation with the hydroxyl group pointing outwards which would allow sufficient binding for detectable catalytic activity. In this instance, the phenol side of caffeate facing towards the binding cavity would have no additional side group on the aromatic ring, as is the case for *p-*coumarate, towards which AnFaeA shows detectable catalytic activity.

The full range of solutions with factors such as orientation, mean binding energy (MBE) as well as the number of solutions within each clustered solution, the latter being a test of robustness of the derived docking, were considered in the simulations (Table 3). While the correct orientation in terms of the proximity to the catalytic serine was found for all five methyl and ethyl hydroxycinnamates (similar to that found by McAuley *et al*.^24^), for MCA and MpCA there was a more favourable alternative docking conformation. This alternative conformation occupied nearly the same space as the catalytic binding orientation but the placement of the aromatic ring was on the opposite side of the binding cavity. These reversed solutions for MCA and MpCA are unlikely to be catalytic as the estimated distance between the catalytic serine and the carbonyl carbon of the ester bond is 6.49 Å compared with 2.40 Å between the serine and carbonyl carbon in the catalytic orientation. Furthermore, the binding energy of MCA in the reversed orientation is 0.85 kcal/mol lower than for the catalytic orientation, and almost twice as represented in robustness (Table 3). Similarly, the reversed orientation for MpCA is 0.91 kcal/mol lower and over 3-fold more represented in the clustering of the docking results (Table 3). In fact, binding energies for the reversed orientations of MpCA and MCA are similar to the binding energies of MSA and MFA in their catalytic orientations. Assuming that the reversed orientations are in fact occurring for MpCA and MCA in the enzyme active site, this could explain the low apparent catalytic rates by AnFaeA towards these substrates. That is, MpCA and MCA occupy non-catalytic orientations at least as often, if not more frequently than catalytic orientations, leading to low apparent affinities for the substrates.

**Table 3:** Mean Binding Energy (MBE) and number of solutions in each solution cluster (N Clusters) for small molecule docking of hydroxycinnamic alkyl and arabinose esters to AnFaeA (PDB: 1UZA).

Ligand	Catalytic Orientation		Reversed Orientation	
	MBE (kcal/mol)	N Clusters	MBE (kcal mol)	N Clusters
Alkyl esters				
MSA^#^	−6.46	15	—	—
MFA	−6.32	9	−5.59	5
EFA	−6.36	13	−5.97	3
MCA^*^	−5.40	5	−6.25	9
MpCA^*^	−5.85	4	−6.76	14
Arabinose esters				
ASA^#^	−7.15	8	—	—
AFA^#^	−5.81	5	—	—
ACA	−5.90	6	−5.72	3
ApCA	−5.69	5	−5.19	2

The lower the MBE the greater the affinity for substrate binding while having a larger number of solutions in the solution cluster indicates that the orientation is robust. *substrates have more favourable binding in a reversed orientation into the catalytic site of AnFaeA. ^#^MSA^9^, ASA and AFA (this study) had no reversed orientations in simulations and are substrates for which AnFaeA shows highest catalytic activity.

**Figure 6:**
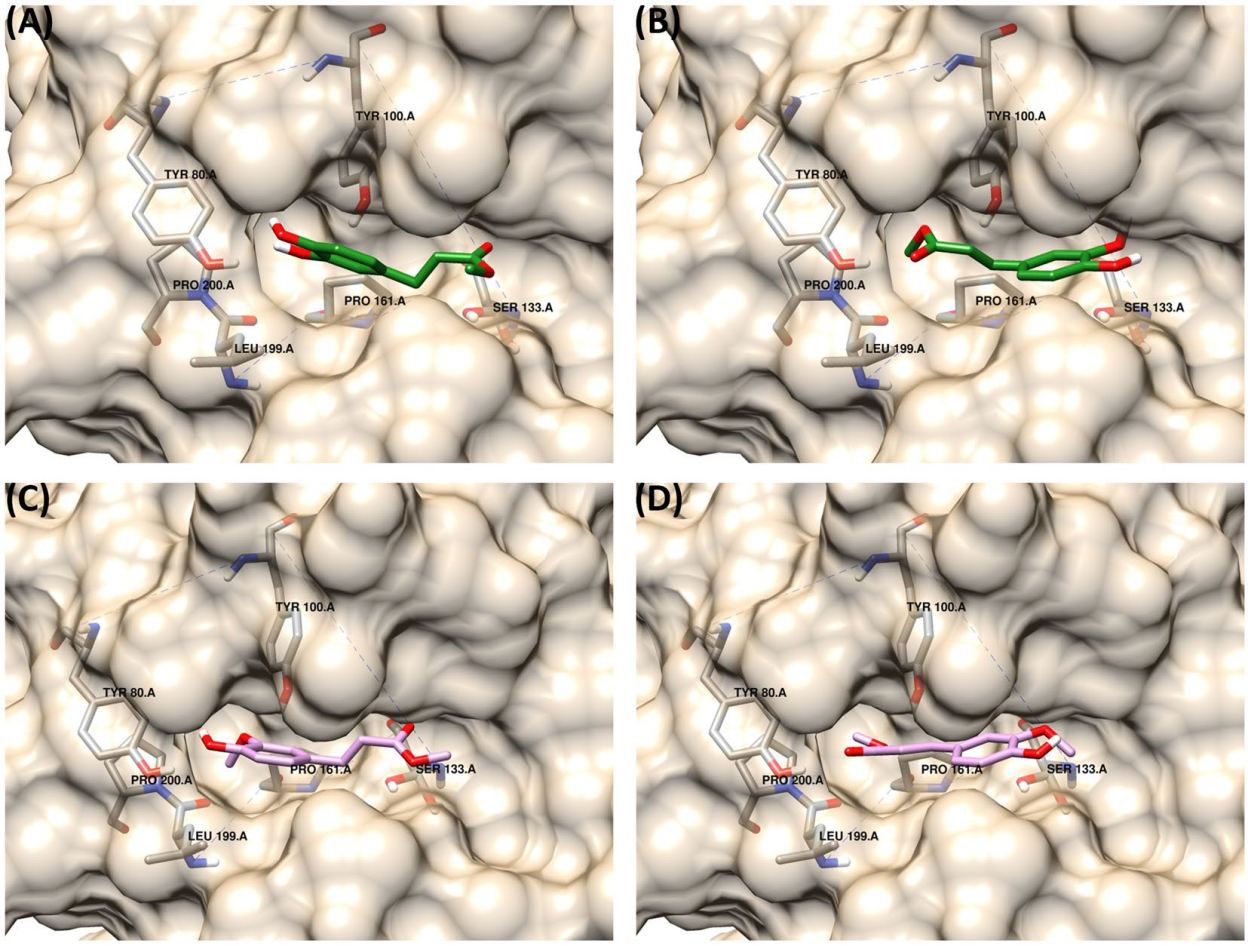
Binding of MCA and MFA into the active site of AnFaeA. (**A**) represents the catalytic orientation of the MCA (**B**) shows an alternative non-catalytic orientation for MCA that is more energetically favourable as the methyl group occupies the hydrophobic hole however this more than doubles the distance from the catalytic serine. For reversed orientation the methyl group occupies the hydrophobic pocket that would normally be occupied by the methoxy side group of substrates like MFA. (**C**) represents the catalytic orientation for MFA while (**D**) represents an alternative non-catalytic orientation for MFA that is also energetically favourable.

When visualised as docked substrates in the active site of AnFaeA, MpCA and MCA show an interesting phenomenon; the hydrophobic pocket that accommodates the methoxy side group of ferulate and sinapate is occupied by the methyl alcohol of MpCA or MCA. The residues that stabilise the 4′-OH of MCA in the catalytic orientation instead stabilise the carbonyl oxygen and residue TYR100 stabilises the ester bond (Fig. 6A and B). That is, the methyl ester bond is accommodated similarly to that of the methoxy residue in MFA or MSA and the distance between the 4′-OH of the phenol ring and the methoxy oxygen in MFA/MSA is a similar to the distance between the carbonyl oxygen and the ester oxygen in MCA and MpCA (i.e. 2.7 Å versus 2.4 Å). Thus, a hydroxycinnamate lacking the methoxy substituents may be represented in both orientations where the methyl ester is a proxy for the methoxy group and occupies this same position. This observation serves as an explanation for the undetectable activity of AnFaeA towards MCA: the substrate’s binding is stabilised in the reversed, non-catalytic orientation. MFA is also seen to dock in two orientations (Fig. 6C and D) similarly to MCA. In the catalytic orientation the methoxy side group on the phenol ring occupies the hydrophobic hole while for the reversed orientation the methyl ester group occupies a highly similar space to that observed for MCA in that orientation. The major difference in MFA compared with MCA is that the catalytic orientation is more energetically favoured than the alternative non-catalytic orientation, indicating that the docking seen in Fig. 6C would be preferable to that of Fig. 6D, compared with Fig. 6B being more preferable than Fig. 6A for MCA. While MpCA does also bind in a reversed orientation at a more favourable energy, its binding energy in the forward direction is lower than that of MCA. Additionally, without the hydroxy group on the phenol ring, MpCA would be less sterically hindered and more readily transition between the active and inactive state. The more favourable binding energy coupled with the absence of the side group would explain the observed very low activity of AnFaeA on MpCA (As low as 0.1 *Umg* ^−1^ protein^25^ for MpCA, compared with 67.2 for MFA), marginally higher than the now observed activity on MCA. A more comprehensive study of the factors that contribute to docking orientation would require the use of molecular dynamics simulation which was outside the scope of this manuscript.

### Docking of arabinose esters to AnFaeA is favourable in the catalytic orientation

**Figure 7:**
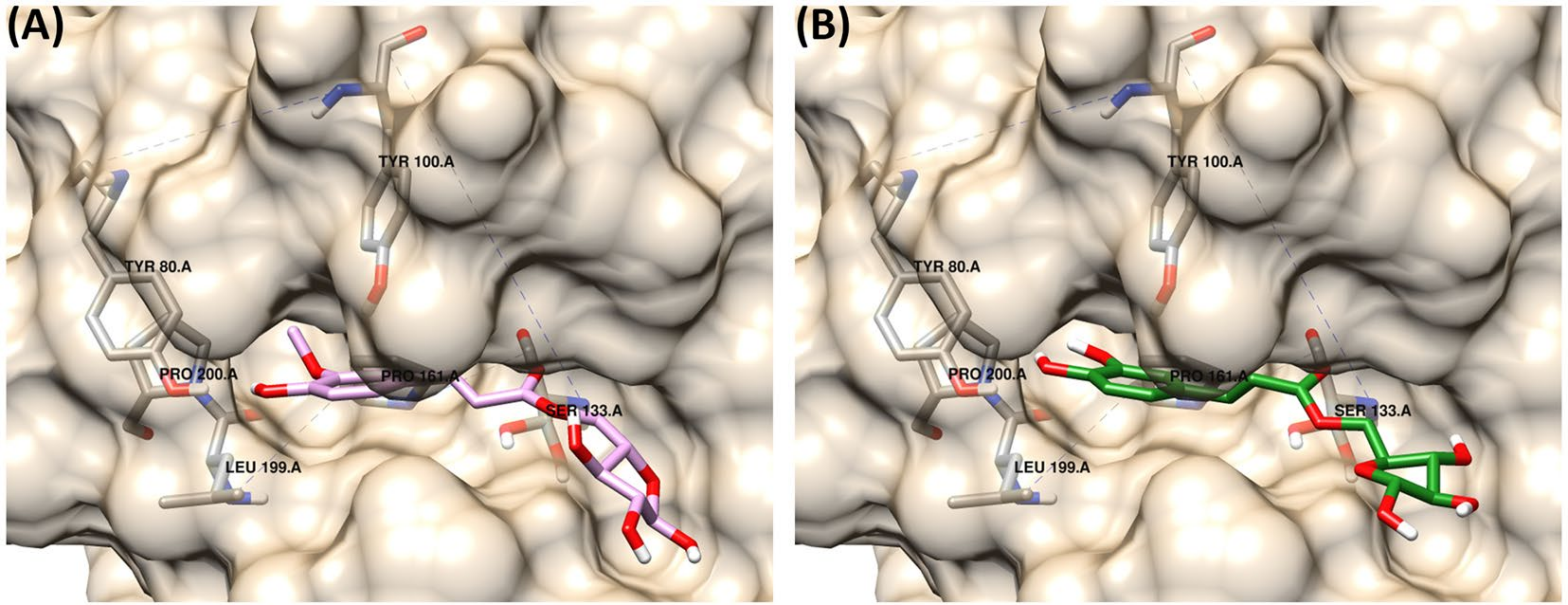
Binding of arabinose hydroxycinnamic esters into the active site of AnFaeA. (**A**) docking of AFA into the active site (**B**) docking of ACA in a catalytic orientation; the arabinose molecule reduces the favourability of the reversed, non-catalytic binding.

To further investigate the effect of the residues in the ester linkage on substrate docking and compare these results to the catalytic activity profile measured with AnFaeA, the alkyl esters were replaced with arabinose in the docking simulations. Arabinose is particularly relevant as enzyme characterisation revealed that AnFaeA was significantly more active on the arabinose esters of ferulate and caffeate than methyl esters. Both AFA and ACA dock favourably in the forward, catalytic orientation (Fig. 7A and B) with binding energies of −5.81 and −5.90 kcal/mol respectively (Table 3). Here, the non-catalytic orientations are not favourable as the larger hydrophilic arabinose residues are unable to dock into the hydrophobic hole and in the reversed orientation the sugar group occupies a large proportion of the binding cavity (data not shown). This orientation diverts the docking position of the ester bond away from the catalytic serine and was the likely reason that no solutions were obtained for AFA in the reversed orientation (as well for ASA). A reversed orientation was identified for ACA but was highly unfavourable and less robust as it was stabilised through one hydrogen bond between THR68 and the hydroxyl on the phenolic ring (data not shown).

**Figure 8:**
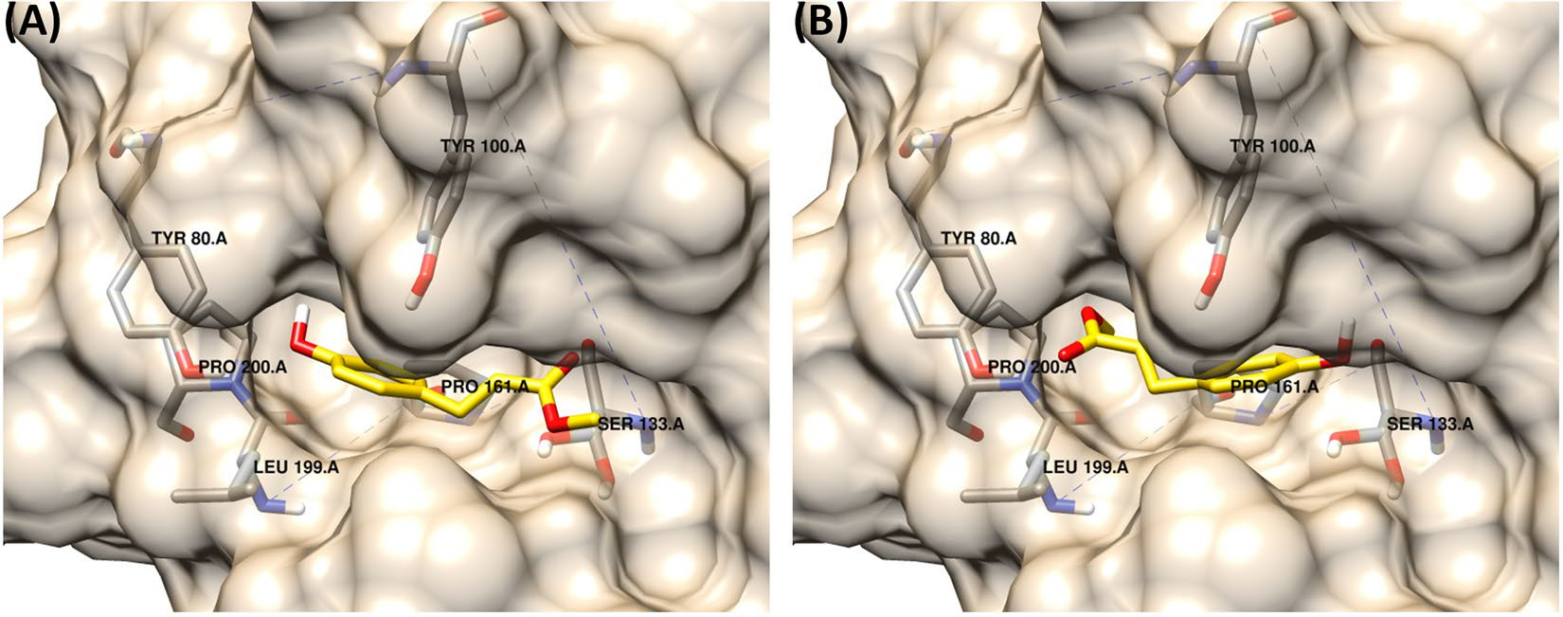
Binding of MpCA into the active site of AnFaeA. (**A**) represents the correct catalytic orientation while (**B**) shows an alternative non-catalytic orientation that is more energetically favourable as the methyl group occupies the hydrophobic hole similar to that observed with MCA.

In this study we replaced a small alkyl ester with a larger, more polar sugar residue and this had a considerable effect on the simulated docking of the esters into the enzyme active site and catalytic activities. These findings support our proposition that the previously determined low activity of AnFaeA toward MCA, and by extension to MpCA, may be due to the more favoured non-catalytic binding orientation in the active site. The docking simulation for MpCA, given in Fig. 8 for the catalytic and non-catalytic orientations, show the molecule is reversed in such a way that the methyl ester occupies the hydrophobic hole that would be occupied by the methoxy residue (of substrates such as MFA or MSA) on the phenolic ring. Replacing the methyl group of MpCA with arabinose (ApCA) again shifts the simulation results favouring the catalytic orientation in MBE and robustness (Table 3).

The docking simulations provide a possible mechanism for the difference in AnFaeA activities toward MCA and ACA: by replacing the small alkyl with the arabinose ester, the reversed orientation is much less favourable, with poorer MBE and clustering (Table 3). The larger arabinose group does not effectively occupy the pocket for the phenolic head preventing its docking and indicates the basis of the reversed, non-catalytic orientation can be heavily attributed to the methyl ester. In particular, the reversed orientation is significantly more poorly represented in the docking simulations when the ester is an arabinose residue, irrespective of which hydroxycinnamic acid is present. The methyl ester of CA binds in an energetically favourable, but non-catalytic manner to AnFaeA and this is supported by the very low measured catalytic activity and lower substrate affinity observed compared with ACA (Table 2). Similarly, an absence of a favourable reversed orientation for AFA in the AnFaeA active site correlates well with its higher affinity and much greater activity compared with MFA.

In this manuscript, we prove through systematic experiments and validation of circumstantial reports (particularity in regards to increased catalytic activity towards FAX, FAXX and other feruloylated arabinose xylan esters^18,19,26,27^) that arabinose hydroxycinnamates, as natural substrates, offer better binding and therefore higher activity. Given this observation, AnFaeA, previously classified as type A, does indeed show activity towards caffeate-type esters that have 3′-hydroxy substitution on the phenolic ring. In revision, the enzyme could well be classed as type C or D FAE with broad activity. This offers an insight into the binding of methyl and arabinose esters to AnFaeA and subsequent catalytic activity, which can in turn be extended to other FAE types. In essence, arabinose based substrates are a more appropriate ligand for determining substrate specificity and thus can aid in the classification of FAE. While by no means are the authors proposing a complete novel classification for FAE, as this is an objective of future work, such as classification system could combine the idea of endo and exolytic FAE activity^28^ with the observation that all FAE could potentially demonstrate broad substrate activity with a preference for moiety but not exclusion. Thus the structural elements of the *β*-clamp and the hydrophobic hole are better rectified with activity profiles. However, the establishment of such a classification system is subject to further investigation of other arabinose esters to further characterised FAE with known crystal structures. *Ad Interim*, this manuscript provides evidence that arabinose esters are superior and therefore a novel classification system can be created in the near future based on these specific substrates.

## Methods

### Enzyme production and purification

Recombinant FAE type A from *A. niger* (AnFaeA) was expressed in *Pichia pastoris* as described previously^28^. For the synthesis of arabinose esters, a type B FAE from *Myceliopththora thermophila* ATCC 42464 (MtFae1a) was expressed in *P. pastoris*, as described previously^20^. The enzymatic preparations were concentrated and exchanged for 100 mM MOPS-NaOH pH 6.0. Protein concentration was determined by the Pierce^TM^ BCA Protein Assay (ThermoFisher Scientific, USA). The FAE purity (%) of the enzyme preparations was determined by SDS-PAGE using a Novex Sharp pre-stained protein standard (Life Technologies, USA), followed by quantification using the JustTLC software (Sweday, Sweden).

### Hydroxycinnamic ester substrates for enzyme reactions

MFA and MCA (99%) were purchased from Alfa Aesar (Germany). Arabinose ferulate and arabinose caffeate (AFA and ACA, respectively) were synthesized through enzymatic transesterification at preparative scale (10 mL) in sealed flasks containing 70:30 v/v 100 mM MOPS-NaOH pH 6.0: dimethyl sulfoxide (DMSO). 50 mM of the donor (104 mg MFA or 97 mg MCA) was diluted in DMSO while 700 mM acceptor (L-arabinose, 1.051 g) and 0.08 mg MtFae1a mL^−1^ were introduced in the form of concentrated stock solutions in buffer. Reactions were carried out at 35 °C for 24 h without agitation.

### Isolation and characterisation of arabinose hydroxycinnamate substrates

Isolation of the arabinose ester from each flask was performed by preparative HPLC on a C18 Luna 5 *μ* column (250 mm × 21.2 mm) (Phenomenex) according to Vafiadi *et al*.^16^. The reaction mixture after evaporation under vacuum and mild heating (50 °C) was diluted in methanol (1 mL). Fractions containing AFA (3.2%, 3.3 mg) or ACA (12.9%, 12.5 mg) were pooled and evaporated. Proton NMR spectroscopy was done in DMSO-*d*6 on a Bruker Ascend Aeon WB 400 spectrometer.

^1^H NMR (DMSO-*d*6, 400 MHz) 5-*O*-(*trans*-feruloyl)-L-arabinofuranose: ***δ*** 9.60 (s, 1H, ArO***H***), 7.57 (dd, 1H, J1 = 4 Hz, J2 = 16 Hz, -C***H***CHCOOR), 7.34–7.32 (m, 1H, Ar***H***), 7.12 (dd, 1H, J1 = 1.6 Hz, J2 = 8.4 Hz, Ar***H***), 6.79 (d, 1H, J1 = 8 Hz, Ar***H***), 6.49 (dd, 1H, J1 = 4 Hz, J2 = 16 Hz, -CHC***H***COOR), 6.26–6.24 (m, 1H, -OCHO***H***), 5.32–5.28 (m, 2H, >CHO***H***), 4.96 (dd, 1H, J1 = 2.8 Hz, J2 = 5.2 Hz, >OC***H***OH), 4.30–4.25 (m, 1H, >C***H***2), 4.12–4.08 (m, 1H, >C***H***2), 4.00 (dt, 1H, J1 = 2.8 Hz, J2 = 6.8 Hz, >CH2C***H***), 3.82 (s, 3H, -OC***H***3), 3.74–3.72 (m, 1H, >C***H***OH), 3.68–3.64 (m, 1H, >C***H***OH).

1H NMR (DMSO-*d*6, 400 MHz) 5-*O*-(*trans*-caffeoyl)-L-arabinofuranose: ***δ*** 7.48 (dd, 1H, J1 = 4 Hz, J2 = 16 Hz, -C***H***CHCOOR), 7.04 (d, 1H, J1 = 2 Hz Ar***H***), 7.01–6.98 (m, 1H, Ar***H***), 6.74 (d, 1H, J1 = 8 Hz, Ar***H***), 6.24 (dd, 1H, J1 = 4 Hz, J2 = 15.6 Hz, -CHC***H***COOR), 4.95 (d, 1H, J1 = 2.8 Hz, >OC***H***OH), 4.32–4.29 (m, 1H, >C***H***2), 4.11–4.06 (m, 1H, >C***H***2), 4.00 (dt, 1H, J1 = 2.8 Hz, J2 = 7.2 Hz, >CH2C***H***), 3.74–3.72 (m, 1H, >C***H***OH), 3.67–3.64 (m, 1H, >C***H***OH).

### Biochemical characterisation of AnFaeA

For the characterization of AnFaeA, stock solutions of substrate (MFA, AFA, MCA, ACA) (5–10 mM) were prepared in DMSO. Each reaction was initialized by introducing enzyme in the reaction mixture in the form of concentrated stock solution in buffer. The AnFaeA activity was assayed on 0.5 mM substrate in MOPS-NaOH 100 mM pH 6.0 using different enzyme loads. Samples were incubated for 10 min at 45 °C without agitation. All reactions were terminated by incubation at 100 °C for 10 min. One unit (1 U) is defined as the amount of enzyme releasing 1 *μ* mol of free acid per minute under the defined conditions. For determining the effect of substrate concentration on the reaction rate, the action of AnFaeA was studied at varying concentration of substrate (0–2 mM) in MOPS-NaOH 100 mM pH 6.0. Samples were incubated for 30 min at 45 °C without agitation. The kinetic constants (V_max_, K_m_) were determined by fitting the Michaelis-Menten equation to the data using nonlinear regression (R^2^ = 0.9784, p < 0.0001). All assays were carried out in duplicate and concomitant with appropriate blanks. There was no hydrolysis observed in the absence of enzyme.

### Analysis of metabolites of enzyme reactions

Quantitative analysis of samples was performed by HPLC on a C18 Nucleosil 100–5 column (250 mm × 4.6 mm) (Macherey Nagel, Germany). Elution was conducted with a linear gradient method using water (solvent A) and acetonitrile (solvent B) at 0.6 mL/min and ambient temperature. Total running time was 20 min during which the following proportions of solvent B were used: 0–15 min 28–72% and 28% 15–20 min. Detection was achieved by a PerkinElmer Flexar UV/VIS detector at 300 nm based on calibration curves prepared using standard solutions of the detected compounds in 1:1 water:acetonitrile.

### Small molecule docking of hydroxycinnamic ester substrates

Docking simulations were conducted as per the earlier report of Suzuki *et al*.^14^ and consistent for all substrates. Here the rigid macromolecule was Chain A of the crystal structure of AnFaeA (PDB: 1UZA). Substrates used were the methyl esters (MSA, MFA, MCA and MpCA), the ethyl ester EFA, and the arabinose 5-O esters (ASA, AFA, ACA and ApCA). All were generated using Avogadro^29^ and structure optimised using UFF.

Briefly, PDB entry 1UZA was first prepared by relaxing side group atoms using the amber suite^30^, keeping backbone atoms stationary. The PDB file was also cleaned for non-standard residues and water molecules were removed. AutoDockTools (ADT)^31^ was used to add polar hydrogen and to assign Gasteiger charges to all atoms within Chain A. A grid box of 80 × 80 × 80, centred on the catalytic serine was used as the search space with a grid spacing of 0.375 Å.

AutoDock (AD) was used for docking via a Lamarckian genetic algorithm^31^ with random starting conformations of the ligand with the maximum number of torsions available. Results were visualised using ADT or Chimera^32^.

### Data Availability

The protein sequences for FAE from *A. niger* (AnFaeA) and from *Myceliopththora thermophila* ATCC 42464 (MtFae1a) are available from NCBI (Accession: O42807.1 & G2QND5.1 respectively). The structure of AnFaeA is available from PDB (1UZA). The hydroxycinnamic ester substrates used in the SMD as well as the output AutoDock log files containing the docked solutions are available on reasonable request, from the corresponding author.
